# Effects of turmeric (Curcuma longa) on oral health

**DOI:** 10.6026/97320630018538

**Published:** 2022-06-30

**Authors:** Vidhya Rekha Umapathy, Bhuminathan Swamikannu, Sumathi Jones, M Kiran, Tanuja Lell, Vinyas Mayasa, Jayamathi Govindaraj

**Affiliations:** 1 Sree Balaji Medical College and Hospital, Chromepet, Chennai 600044, India

**Keywords:** Turmeric, curcumin, pharmacological actions, dentistry

## Abstract

The turmeric plant was used in ancient medicine to cure a wide range of diseases, including cough, diabetes, and liver disease. Data shows that the principal chemical component of turmeric, curcumin, has a variety of beneficial effects on the body. Therefore, it is of interest to document data on the therapeutic activities of turmeric, including its extracts and possible medical uses, as well as its oral and dental uses and a safety assessment of those uses. Curcumin, the most pure form of turmeric, has shown promise in dentistry.

## Background:

The most frequent ingredient in Indian cuisine is turmeric, commonly known as "Indian Saffron" [1-2]. It has been known for a long time that it can help treat a wide range of illnesses at home, and it is now being used more and more in advanced medical field. Curcumin comes from the root of the plant Curcuma longa Linn [3]. According to Ayurveda, turmeric can be used to treat eye infections, burns, acne, and skin diseases, as well as a bitter digestive and carminative [4]. Antioxidant, anti-inflammatory, antibacterial, anti carcinogenic, and antimutagenic activities are possessed by this substance. More than 100 studies have been done on how natural substances affect human health, disease prevention, and treatment. Polyphenols derived from natural sources represent one of the most significant groups. Curcuminoids are phenolic chemicals generated from curcuma spp. root systems (Zingiberaceae). The powdered form of C. longa's rhizome is abundant in curcuminoids. Recent efforts in western medicine to use natural and complementary therapies have brought this old cure to the attention of scientists. Research shows that curcumin has many health benefits, such as anti-inflammatory, anti - oxidant, anti - carcinogenic and chemotherapeutic actions. Both lab-grown cells and animal models have shown that these activities work, which opens the door for ongoing clinical trials in people. This review is a summary of studies that describe curcumin's effects, how it works, and its chemical and clinical properties [4].

## Mechanism of action of turmeric:

Curcumin is poorly bioavailable orally. Due to poor intestinal absorption, a fast metabolic rate, and rapid systemic clearance, its oral bioavailability is low. 40 to 85 percent of an oral dose of curcumin is eliminated unaltered from the digestive tract. Curcumin is combined with bormelain to enhance its bioavailability and anti-inflammatory action [5].

## Turmeric's medicinal and pharmacological qualities:

### Effects on inflammation:

Significant anti-inflammatory effects are provided by the volatile oils and curcumin found in Curcuma longa. Acute inflammation treated with oral curcumin was found to be just as effective as chronic inflammation treated with cortisone or phenylbutazone. The inflammatory swelling caused by Freund's adjuvant-induced arthritis in rats was significantly reduced when Curcuma longa was supplied orally in comparison to controls. A study in monkeys found that curcumin reduced the aggregation of neutrophils linked to inflammation. Prostaglandin production from arachidonic acid and neutrophil activity in inflammatory situations can be blocked by Curcuma longa, which has anti-inflammatory properties. Additionally, curcumin can be applied topically in order to alleviate the symptoms of inflammatory skin conditions and allergies. In 2009, R. Vivek did a study to determine the anti-inflammatory efficacy of cyclodextrin (CD) complex of curcumin for the treatment of inflammatory bowel disease (IBD) in a rat model of colitis. Curcumin has a stronger affinity for hydroxypropyl - CD (HPCD) than for other CDs, as demonstrated by in vitro dissolution studies. Curcumin's anti-angiogenic and anti-inflammatory effect in chick embryos and rats was further investigated utilising the HPCD complex of curcumin, and it was found that the CD of curcumin considerably reduced the degree of colitis caused by DSS (dextran sulphate solution) administration. Patients with inflammatory bowel disease (IBD) may benefit from curcumin, a naturally occurring dietary compound [6].

### Antibacterial property of curcumin:

Curcuma longa variations kasur, Faisalabad, and Bannu's essential oil and crude extracts of curcuminoides were used in a 2010 study by Shaguftanaz to explore the antibacterial activities of the various Curcuma longa variants. Curcuminoids were extracted with ethanol, and the oil exhibited zone of inhibition against all tested bacterial strains. In comparison to the other two turmeric kinds, the Kasur variety inhibited the growth of all tested bacterial strains the most effectively [7].

### Anti-fungal action of curcumin:

Curcumin and components produced from the rhizomes of Curcuma longa shown antifungal efficacy against a variety of plant-pathogenic fungi. The reactions differed depending on the examined pathogen. Curcumin had fungicidal activity comparable to that of the fungicide chlorothalonil [8].

### Anti oxidant property of curcuminoides:

Curcuma longa's curcuminoids were tested for biological activity by Simay Cikriker and Erkan Mozioglu in 2008. With the CUPRAC approach, the antioxidant activity of curcumin and turmeric was also examined; turmeric extracts and pure curcumin showed modest antibacterial and antifungal activity, in addition to having highly good antioxidant capabilities [9].

### Curcumin's hepatoprotective properties:

Turmeric's hepatoprotective properties stem mostly from its antioxidant properties and ability to suppress the generation of pro-inflammatory cytokines.When administered to ducklings infected with Aspergillus parasiti custurmeric extract suppressed aflatoxin formation by 90 percent.

## Curcumin's impact on macrophages:

Macrophages are a very important part of the immune system. They help the body fight off foreign proteins and then get rid of them successfully. Curcumin was given to the blood macrophages of nine people: six people with Alzheimer's disease and three healthy people who served as controls. Then, beta amyloid was introduced. When macrophages of Alzheimer's patients were treated with curcumin, plaque was taken up and digested better. Because of this, curcumin may help the immune system get rid of amyloid protein [10].

## Liver diseases:

Turmeric has a positive effect on the liver. Increased herb and food consumption in the spring can strengthen the liver. Similar liver-protective chemicals are found in turmeric, milk thistle, and artichoke plants. It is useful in treating liver disorders such as hepatitis, cirrhosis, and jaundice, as it is supposed to shrink enlarged hepatic ducts. In 2008, Kwon-ll So did a study to find out what effect taking curcumin had on the blood glucose, plasma insulin, and enzyme activities related to glucose homeostasis in diabetic mice. Curcumin seemed to be a potential glucose-lowering agent and antioxidant in db/db mice with type 2 diabetes, but it didn't have any effect on db/plus mice without diabetes [11].

## Iron chelator:

Yan Jiao did a study in 2005 to figure out how iron chelation affects the biological effects of curcumin. He did this by looking at the effect of curcumin on transferrin receptor 1, a protein that gets stronger when there isn't enough iron, and the ability of curcumin to turn on iron regulatory proteins (IRPs). Transferrin receptor 1 and active IRP, which are both signs of low iron, went up when curcumin was given. The amount of ferritin protein in the liver of mice whose diets included curcumin decreased. These findings imply that iron chelation might be an alternative mechanism of action of curcumin [12].

## Anti cancer agent:

Due in part to its antioxidant action, turmeric shows great potential as a cancer treatment. Recent studies have connected the regular consumption of turmeric to lower incidences of breast, colon, lung, and prostate cancer. According to laboratory testing, curcumin may inhibit the formation of tumours and decrease the spread of cancer cells. Curcumin's efficacy in patients with advanced pancreatic cancer is now being evaluated in clinical trials. Additionally, curcumin is frequently prescribed to protect healthy cells from the damaging effects of radiation and chemotherapy without diminishing the efficacy of these treatments [13].

## Therapeutic applications in dentistry:

### Turmeric and oral health:

Numerous medical disorders are treated with turmeric, which is also used in dentistry. Its anti-inflammatory properties aid in the treatment of discomfort, gingivitis, and periodontitis. Additionally, it is employed as a colouring agent in pit-and-fissure sealant and dental plaque detecting systems. Its chemo preventive effect is also helpful for the treatment of precancerous lesions and disorders in the mouth [14].

### Dental inflammatory conditions:

As a mouth rinse, boiling 5 grams of turmeric powder with two cloves and two dried guava leaves in 200 grams of water will quickly reduce inflammation. On painful teeth, massage roasted, ground turmeric can also relieve pain and swelling. Using a paste of 1 tsp of turmeric, 12 tsp of salt, and 12 tsp of mustard oil twice day can reduce gingivitis and periodontitis [15]. Yukie et al. (2014) discovered that C. longa containing toothpaste lowers gingivitis and mild periodontitis [16].

### Dental plaque detection:

Plaque on the teeth is typically invisible to the naked eye since it is typically colourless. In order to detect plaque, turmeric might be employed. It discolours plaque yellow, facilitating its detection [17]. The dental plaque detection system comprises a dental plaque staining agent containing at least one agent selected from the yellow pigment of beni-koji, turmeric extracts, and curcumin; and a light-emitting device that sends light with a wavelength between 250 and 500 nm to an object in the mouth to which the dental plaque staining agent is attached [18].

### Subgingiva lirrigant:

Suhag et al. [19] and Gottumukkala et al. [20] found that a 1 percent curcumin solution could be utilised as a subgingiva lirrigant since it lowers inflammation. Compared to chlorhexidine and saline, the average probing pocket depth of turmeric is less.

### Irrigant for endodontics:

Turmeric can be used to treat infected root canals because it has antibacterial properties and is easy to get, cheap, and has other biological uses. Studies have shown that turmeric can kill bacteria that cause endodontic pathogens and could be used as an endodontic irrigator or an intracanal medication [21].

### Local drug distribution system:

In addition to scaling and root planing, 2 percent turmeric gel can be used as a drug - delivery method to treat periodontitis. This reduces pocket depth and increases clinical attachment levels. Data is known on turmeric's potential as a local medication delivery method [22].

### Recurrent aphthous stomatitis:

Recurrent aphthous stomatitis (RAS) would be an inflammatory disease that affects the mucosa of the mouth. Its cause is unknown. About 20% of the population is affected by RAS at a certain point in life. The illness mostly affects non keratinized mucosal surfaces and is characterised by the recurrence and healing of single or several painful ulcers. Ulcers are accompanied by a "prodrome" that lasts about 24 to 48 hours and is marked by localised burning or pain [26]. It was discovered that turmeric reduces the severity of pain and the size of aphthous ulcers [23].

### Precancerous lesions and conditions:

By virtue of its antioxidant properties and deoxyribonucleic acid-protective processes, turmeric is a beneficial therapy for precancerous lesions and conditions. It enhances serum and salivary vitamin C and vitamin E levels in leukoplakia, lichen planus, and oral submucous fibrosis [24]. Turmeric is an effective, easily accessible, and non-invasive treatment for oral submucous fibrosis, and its use significantly reduces burning sensation [25]. Curcumin doses as high as 6,000 mg per day are useful in reducing oral lichen planus symptoms in individuals.

### Getting rid of dental issues:

A mouth rinse with turmeric water (prepared by boiling 5 grammes of turmeric powder, two cloves, and two dried guava leaves in 200 grams of water) provides immediate relief. Using roasted ground turmeric to massage sore teeth removes pain and swelling. Brushing your teeth after using a paste made from burnt turmeric and bishop's weed seed strengthens your gums and teeth. Gingivitis and periodontitis can be relieved using a paste made of 1 teaspoon turmeric, 12 teaspoons salt, and 12 teaspoons mustard oil. Twice daily, brush your teeth with this paste [26].

### Periodontal problems:

To treat gingivitis and periodontitis, a paste made of 1 teaspoon turmeric, 12 teaspoons salt, and 12 teaspoons mustard oil is applied topically. Using this paste on your teeth and gums twice a day is recommended [26].

### Mouth wash:

Around 100 patients were selected at random for a study by Das et al. They measured the gingival index and the plaque index three times over the course of the study: at 0, 14, and 21 days. Chelxidine gluconate and turmeric mouthwash were found to be useful in conjunction with mechanical plaque management procedures for the prevention of plaque and gingiva. The turmeric mouthwash, which is created by dissolving 10 mg of curcumin extract in 100 ml of distilled water with 0.005% peppermint oil and a pH of 4, is as efficient as being the most regularly used chlorhexidine mouth wash. However, chlorhexidine gluconate has been found to be more effective in terms of its antiplaque properties. The reported impact of turmeric may be due to its anti-inflammatory properties. Reductions in the overall number of microorganisms were seen in both groups [27].

### Curcumin's effect on human gingival fibroblasts:

Curcumin boosted the proliferation of regular human fibroblasts and micro vascular endothelial cells (hMVEC) at lower doses, but it reduced at higher levels [28]. Curcumin-treated hPGF cells displayed maximal and considerable apoptosis at 75 M, as well as morphologic abnormalities in basal cell carcinoma cells treated with 50nM curcumin, including cell shrinkage, removal of microvilli, and emergence of membrane blebbing, according to other investigators [29].

### Sealant for pits and fissures:

The use of a coloured pit and fissure sealant to tooth surfaces has been found to aid in the prevention or reduction of dental caries. A polymerizable resin system containing acrylic monomer and at least one colourant from the group of Annatto extract, turmeric extract, and L-Apo-8-Carotenal can be used to produce this sealant [30].

## General turmeric health benefits:

Wounds and burns can be treated with this natural antiseptic and antibacterial substance. When consumed with cauliflower, it has been shown to protect prostate cancer and limit its growth. As a preventative measure; it may also cause the death of cancerous cells. Breast cancer in mice was halted from spreading to the lungs, and childhood leukaemia risk was lowered. Amyloid plaque development in the brain may be eliminated, preventing Alzheimer's disease progression and cancer metastasis. A potent natural anti-inflammatory; it has the same anti-inflammatory benefits of pharmaceuticals but none of the negative side effects. Multiple sclerosis has been shown to be delayed in mice while using this treatment. Curcumin, a naturally occurring painkiller and cox-2 inhibitor, may help with fat metabolism and weight loss. It has been used in Chinese medicine for millennia as a treatment for depression. The anti-inflammatory properties of this herb make it an effective treatment for arthritic and rheumatic conditions. Currently, researchers are investigating whether turmeric can help treat multiple myeloma by inhibiting the growth of new blood vessels in malignancies. It aids in the regeneration of injured skin and speeds up the healing process when a wound has been damaged. The treatment of psoriasis and other inflammatory skin conditions may be helped by this method [30].

## Future challenges:

Due to its poor absorption, rapid metabolism of curcumin in the intestines, and fast departure from body, producing curcumin for medicinal use is a major challenge. Furthermore, there is insufficient data to assess its safety at greater doses. Curcumin bioavailability and perceived toxicity are being vigorously investigated using nanotechnology-based new methodologies around the world.

## Conclusion:

Turmeric anti-inflammatory, antibacterial, and anticancer properties, along with its multiple therapeutic uses, could be utilised to address a wide variety of illnesses, not just within dentistry, as well as in general oral health. More research is needed to prove its specific purpose, appropriate doses, and other pharmacokinetic features. With such a diverse range of therapeutic applications, "turmeric" can be viewed as a future windfall for dental health.
